# PROTECTIVE RESILIENCE INDEX FOR SUCCESSFUL AGING IN MUSCULOSKELETAL PAIN (PRISM): PRELIMINARY FINDINGS

**DOI:** 10.1093/geroni/igac059.427

**Published:** 2022-12-20

**Authors:** Amber Brooks, Kimberly Sibille, Emily Bartley, Cynthia Garvan, Angela Mickle

**Affiliations:** Wake Forest School of Medicine, Winston-Salem, North Carolina, United States; University of Florida, Gainesville, Florida, United States; Department of Community Dentistry &Behavioral Science, Gainsville, Florida, United States; Department of Anesthesiology, Gainesville, Florida, United States; Physical Medicine and Rehabilitation, Gainesville, Florida, United States

## Abstract

Chronic musculoskeletal pain is a highly prevalent condition in older adults and is associated with a decrease in function, biological dysregulation and cellular aging, and increased risk of morbidity and mortality. Our previous work shows that a resilience index comprised of measures of recognized biobehavioral and psychosocial protective factors based on clinically validated norms (waist hip ratio, tobacco use, social support, perceived stress, optimism, positive affect, negative affect-low, active coping) was positively associated with telomere length, lower levels of clinical pain and pain-related functional decline which, also correlated with pain-related brain structure. We will report the initial findings from our multi-centered, NIA RCCN funded study, Protective Resilience Index for Successful Aging in Musculoskeletal Pain (PRISM), which seeks to refine and validate a resilience index that is predictive of biological and pain-related outcomes. This work will lay the foundation for a resilience index that can be used in everyday clinical practice.

